# Impact of Center-related Characteristics and Macroeconomic Factors on the Outcome of Adult Patients With Acute Lymphoblastic Leukemia Treated With Pediatric-inspired Protocols

**DOI:** 10.1097/HS9.0000000000000810

**Published:** 2022-12-23

**Authors:** Pere Barba, Mireia Morgades, Pau Montesinos, Jose Gonzalez-Campos, Anna Torrent, Cristina Gil, Teresa Bernal, Mar Tormo, Santiago Mercadal, Sandra Novoa, Irene García-Cadenas, M. Paz Queipo de Llano, Marta Cervera, Rosa Coll, Arancha Bermudez, M. Luz Amigo, Silvia Monsalvo, Jordi Esteve, Raimundo Garcia-Boyero, Andres Novo, Jesús Maria Hernandez Rivas, Antonia Cladera, Pilar Martinez-Sanchez, Josefina Serrano, Maria Teresa Artola, Beatriz Soria, Eugenia Abella, Ferran Vall-Llovera, Juan Bergua, Pilar Herrera, Daniel Barrios, Josep Maria Ribera

**Affiliations:** 1 Hematology Department, Hospital Universitari Vall d’Hebron, Barcelona, Spain; 2 Hematology Department, Institut Català d’Oncologia-Hospital Germans Trias i Pujol, Josep Carreras Research Institute, Badalona, Universitat Autònoma de Barcelona, Spain; 3 Hematology Department, Hospital Universitari i Politècnic La Fe, Valencia, Spain; 4 Hematology Department, Hospital Universitario Virgen del Rocío, Sevilla, Spain; 5 Hematology Department, Hospital General Universitario de Alicante, Spain; 6 Hematology Department, Hospital Universitario Central de Asturias, Oviedo, Spain; 7 Hematology Department, Hospital Clínico Universitario de Valencia, Spain; 8 Hematology Department, Institut Català d’Oncologia-Hospital Duran i Reynals, L’Hospitalet de Llobregat, Spain; 9 Hematology Department, Hospital de la Santa Creu i Sant Pau, Barcelona, Spain; 10 Hematology Department, Hospital Universitario Virgen de la Victoria, Málaga, Spain; 11 Hematology Department, Institut Català d’Oncologia-Hospital Joan XXIII, Tarragona, Spain; 12 Hematology Department, Institut Català d’Oncologia-Hospital Josep Trueta, Girona, Spain; 13 Hematology Department, Hospital Universitario Marqués de Valdecilla, Santander, Spain; 14 Hematology Department, Hospital General Universitario Morales Meseguer, Murcia, Spain; 15 Hematology Department, Hospital General Universitario Gregorio Marañón, Madrid, Spain; 16 Hematology Department, Hospital Clínic de Barcelona, IDIBAPS, Universitat de Barcelona, Spain; 17 Hematology Department, Hospital General Universitario de Castellón, Castellón de la Plana, Spain; 18 Hematology Department, Hospital Universitari Son Espases, Palma de Mallorca, Spain; 19 Hematology Department, IBSAL, IBMCC, Centro de Investigación del Cáncer, CIBERONC, Universidad de Salamanca-CSIC, Hospital Universitario de Salamanca, Spain; 20 Hematology Department, Hospital Universitari Son Llàtzer, Palma de Mallorca, Spain; 21 Hematology Department, Hospital Universitario 12 de Octubre, Madrid, Spain; 22 Hospital Universitario Reina Sofía, IMIBIC, Córdoba, Spain; 23 Hematology Department, Hospital Universitario de Donostia, Spain; 24 Hematology Department, Hospital Universitario de Canarias, Santa Cruz de Tenerife, Spain; 25 Hematology Department, Hospital del Mar, Barcelona, Spain; 26 Hematology Department, Hospital Universitari Mútua de Terrassa, Spain; 27 Hematology Department, Hospital San Pedro de Alcántara, Cáceres, Spain; 28 Hematology Department, Hospital Universitario Ramón y Cajal, Madrid, Spain; 29 Hematology Department, Hospital Regional Universitario de Málaga, Spain

Diagnosis and treatment of hematological cancers is usually provided in many healthcare facilities including large but also middle size centers.^1^ Providing cancer care in local institutions might be advantageous for patients and caregivers in terms of financial burden and quality of life. However, it might carry potential risks derived of the limited experience of smaller centers and differences in accessibility to complex therapies including allogeneic hematopoietic cell transplantation (allo-HCT) and chimeric antigen receptor (CAR) T-cells. These risks might be especially relevant in infrequent cancers as adult acute lymphoblastic leukemia (ALL).

In most European countries, ALL treatment protocols are based on pediatric-inspired regimens which include a large number of immune-chemotherapeutic agents and several key decision points to allocate patients to distinctive treatment arms based on genetics and treatment response.^3,4^ Several patient and disease characteristics have been identified as prognostic factors for outcomes including age, white blood cell count (WBC) at diagnosis, central nervous system (CNS) infiltration, clearance of measurable residual disease (MRD),^4^ and disease genetics.^5–7^ The outcome of patients with ALL may also depend on external factors including center experience, access to cellular therapies and economic variables.^8–10^ The impact of these center-related and macroeconomic variables on the outcome of patients has been scarcely studied. Thus, the aim of this study was to analyze the potential impact of center-related and macroeconomic variables on the outcome of newly diagnosed adult ALL patients included in 4 consecutive trials of the Spanish Program for Treatment of Hematological Malignancies (PETHEMA) Group.

Patients with Philadelphia chromosome (Ph) positive or negative ALL enrolled in one of the 4 consecutive protocols of the PETHEMA group by Spanish institutions adhered to the public health system from 2003 to 2018 were included in this study. The 4 protocols have been closed and reported elsewhere.^4,11–13^ Centralized analysis of MRD was performed in 5 centers in the ALL-AR-03 and in a single institution in the ALL-HR-11 trial. The PH-08 protocol included adults with newly diagnosed Ph-positive ALL up to the age of 60 years. Patients received an induction therapy with daunorubicin, vincristine, and steroids in combination with imatinib 600 mg/d followed by a 12-week consolidation chemotherapy based on alternated cycles of high-dose methotrexate and cytarabine in combination with imatinib. Allo-HCT was offered to all fit patients with a suitable donor.

Treatment protocols used in the ALL-RE-2008, ALL-AR-03, and ALL-HR-11 trials were pediatric-inspired.^4,11–13^ The ALL-RE-2008 trial included intermediate risk patients based on age (<30 years), WBC count (<25,000 cells/μL), and cytogenetics. The ALL-AR-03 and ALL-HR-11 protocols included high-risk patients up to the age of 60 years diagnosed from 2003 to 2011 and 2011 to 2019, respectively. In both trials, bone marrow MRD assessment by flow cytometry was performed at the end of induction (week 5) and at the end of the third consolidation cycle (weeks 16–18). Only patients with slow clearance of MRD in both trials were allocated to allo-HCT, while patients with good MRD clearance continued with chemotherapy for up to 2 years.

Clinical variables analyzed in this study included age, gender, ECOG performance status, WBC, CNS infiltration, precursor lineage (B or T) presence or absence of Ph and treatment period (2003–2010 versus 2011–2018). The Allo-HCT center was defined as centers having authorization by the Spanish government to perform allo-HCT in the same institution where the patient was treated for the ALL. The Allo-HCT center in the same province was defined as having a designated allo-HCT center in the same province where the patient was treated. Reported ALL referred to the number of ALL patients reported to the PETHEMA database by a particular center and served as a surrogate marker of center experience in treating ALL. Nine centers reporting at least 30 ALL patients each and around half of the patients together in this data set were considered as “experienced centers.” Protocol deviation center referred to centers with identified protocol deviations in key treatment decisions (allo-HCT versus chemotherapy allocation or autologous HCT instead of allo-HCT when not indicated in the protocol) in at least 5% of the patients.

Other demographic and macroeconomic variables are self-explanatory and listed in Table 1. Demographic and economic variables were obtained from the Spanish Government (See footnote in Table 1).

**Table 1:** Patients, Disease, Center, and Macroeconomic Characteristics

Characteristic		N = 816
Age, y, median (range)		35.53 (15; 60)
Gender, n (%)	Male	475 (58)
	Female	341 (42)
ECOG PS, n (%)	0–1	648/772 (84)
	2–3	124/772 (16)
WBC, ×10^9^/L, median (range)		16 (0; 842)
CNS infiltration, n (%)	No	707/769 (92)
	Yes	62/769 (8)
Ph+ ALL, n (%)	No	688 (84)
	Yes	128 (16)
Precursor phenotype, n (%)	B	603/803 (75)
	T	200/803 (25)
Treatment period, n (%)	2003–2010	341 (42)
	2011–2018	475 (58)
Protocol, n (%)	ALL-AR-03	323 (40)
	ALL-RE-08	86 (10)
	PH-08	128 (16)
	ALL-HR-11	279 (34)
Allo-HCT center, n (%)	No	292 (36)
	Yes	524 (64)
Allo-HCT center in the same province, n (%)	No	168/292 (57)
	Yes	124/292 (43)
ALL cases reported to PETHEMA, median (range)		30 (1; 74)
Protocol deviation in ≥5% of reported patients from this center, n (%)	No	445 (55)
	Yes	371 (45)
Beds in the hospital^a^, median (range)		832 (248; 1525)
City population^a^, median (range)		409,661 [29,288; 3,223,334]
AC population^a^, median (range)		6,578,079 [580,229; 8,384,408]
Relative Health investment GDP^a^ (%), median (range)		6.4 [3.9; 9.5]
Health investment/inhabitant €^a^, median (range)		1,312 [1,090; 1,631]
GDP capita/AC, median (range)^a^		22,700 [17,554; 33,824]

a Source: Ministry of Health and Social Services (https://www.sanidad.gob.es/en/estadEstudios/estadisticas/sisInfSanSNS/tablasEstadisticas/InfAnSNS.htm) and Spanish National Statistics Institute (https://www.ine.es/dyngs/INEbase/en/categoria.htm?c=Estadistica_P&cid=1254734710984).

AC = autonomous community; Allo-HCT = allogeneic hematopoietic cell transplantation; CNS = central neurological system; ECOG PS = Eastern Cooperative Oncology Group Performance Status; GDP = growth domestic product; Ph = Philadelphia chromosome; WBC = white blood cell count.

Clinical endpoints included overall survival (OS), disease-free survival (DFS), cumulative incidence of relapse (CIR), and nonrelapse mortality (NRM) were defined as previously described.^11^ Infection-related mortality (IRM) was considered an exploratory endpoint and was defined as patients after the first CR dying of infectious causes during ALL treatment or after allo-HCT without previous ALL relapse.

All numerical variables were summarized and categorized by medians. Spearman’s rank coefficient or median test was used to analyze correlations between factors. OS and DFS curves were plotted by the Kaplan–Meier method and compared by the log-rank test. CIR, NRM, and IRM were estimated using cumulative incidence functions by competing risks analysis, and the Gray test was used for comparisons.^14^ The effects of center-related and macroeconomic indicators on OS and DFS were analyzed using Cox proportional hazards regression models, and the Fine and Gray model was used for analyzing these effects on CIR and NRM.^15^ For each main effect, a separate multivariable model was built, adjusted for other potential risk factors (listed in table 1). All statistical analyses were performed using SPSS (v.24) and R (v.4.1.0). Two-sided values of *P* < 0.05 were considered statistically significant.

Eight hundred sixteen patients were included in the study. Main characteristics at diagnosis appear in Table 1. Median follow-up for patients alive was 3 years (range 0.1–9.5). Two-hundred twenty-four (38%) of the evaluable patients received allo-HCT in the first CR. In line with protocol recommendations, allo-HCT in CR1 ranged from 94% of the patients in the PH-08 trial to 6% patients in the ALL-RE-08 trial. The remaining patients were allocated to further chemotherapy consolidation and maintenance regimens according to each protocol.

Fifty-five centers in 40 different cities located in 15 Spanish Autonomous Communities included at least 1 patient in one of the 4 protocols. Twenty-three (42%) centers were accredited for allo-HCT and 47% of nontransplant institution had an allo-HCT center in the same province. Median number of ALL reported by center to PETHEMA database during the study period was 10 (interquatile range 4–20). Approximately half of the patients (n=424, 52%) were reported by ten centers which were considered *experienced centers* for this study. Patient characteristics were similar between experienced and less-experienced centers, except for treatment period (Suppl.Table S1). Twenty centers (36%) had at least a major deviation in more than 5% of their reported patients. Details on center-related and macroeconomic variables are summarized in Table 1.

As expected, there were some associations among center-related and macroeconomic variables. Hence, being a transplant center was correlated with the number of ALL cases reported (*P* = 0.002), the number of beds in the hospital (*P* < 0.001), and with major protocol deviations (*P* = 0.039). Health investment relative to GDP was inversely correlated with the number of inhabitants in the Autonomous Community (*P* = 0.032) and directly associated with its GDP *per capita* (*P* = 0.001). Other explored associations are listed in Suppl. Table S2.

Probability of OS at 5 years for the whole cohort was 46% (95% confidence interval [CI] 42-50). Variables associated with lower OS in the univariable analysis were older age, higher ECOG PS score, higher WBC at diagnosis, Ph-negative ALL and diagnosis in the earlier period (Suppl. Figure S1). Probability of DFS at 5 years for the whole cohort was 43% (95% CI: 38-47). In the univariable analysis, ECOG PS > 1, higher WBC at diagnosis, Ph-negative ALL, and earlier diagnosis were associated with worse DFS (Suppl. Table S3). In the multivariable model adjusted for macroeconomic variables, none of the center-, region- and economic-related variables was associated with lower OS or DFS (Table 2; Suppl. Figure S2).

**Table 2:** Multivariable Adjusted Model for Center-related and Macroeconomic Variables for Patients Outcomes

Factor	N	OS, HR (IC 95%)	*P*	N	DFS, HR (IC 95%)	*P*	N	CIR, HR (IC 95%)	*P*	N	NRM, HR (IC 95%)	*P*
Allo-HCT center												
No	285	1	0.803	257	1	0.475	274	1	0.450	259	1	0.610
Yes	488	1.029 (0.822;1.288)		442	1.087 (0.864; 1.368)		462	1.111 (0.847; 1.456)		449	1.114 (0.736; 1.688)	
ALL reported cases												
≤30	407	1	0.543	370	1	0.623	394	1	0.320	374	1	0.930
>30	366	1.069 (0.862; 1.326)		329	1.056 (0.850; 1.313)		342	1.137 (0.882; 1.465)		334	0.983 (0.664; 1.457)	
Number of beds in the hospital												
>824	384	1	0.707	346	1	0.504	359	1	0.670	353	1	0.640
≤824	389	1.042 (0.839; 1.295)		353	1.078 (0.865; 1.342)		377	1.057 (0.818; 1.365)		355	0.909 (0.612; 1.352)	
Region population												
≤6,578,079	442	1	0.302	406	1	0.450	429	1	0.210	410	1	0.930
>6,578,079	331	1.122 (0.901; 1.398)		293	1.090 (0.872; 1.363)		307	1.177 (0.913; 1.517)		298	1.019 (0.681; 1.524)	
City population												
≤409,661	396	1	0.446	354	1	0.176	368	1	0.010	360	1	0.240
>409,661	377	1.087 (0.878: 1.345)		345	1.162 (0.935; 1.444)		368	1.399 (1.084; 1.804)		348	0.792 (0.537; 1.169)	
Region GDP per capita (€)												
>22,700	380	1	0.690	341	1	0.660	355	1	0.310	344	1	0.930
≤22,700	393	1.045 (0.842; 1.297)		358	1.050 (0.844; 1.306)		381	1.143 (0.884; 1.477)		364	0.982 (0.660; 1.462)	
Health investment (%)												
≥6.4%	419	1	0.919	380	1	0.859	403	1	0.660	386	1	0.850
<6.4%	354	1.011 (0.814; 1.256)		319	1.020 (0.819; 1.270)		333	1.060 (0.820; 1.371)		322	1.038 (0.670; 1.547)	
Health investment (per capita)												
≥1312	393	1	0.766	350	1	0.963	362	1	0.460	356	1	0.440
<1312	380	0.968 (0.779; 1.202)		349	0.995 (0.799; 1.238)		374	1.100 (0.582; 1.418)		352	0.855 (0.576; 1.270)	

AC = autonomous community; Allo-HCT = allogeneic hematopoietic cell transplantation; CNS = central neurological system; CIR = cumulative incidence of relapse; DFS = disease-free survival; ECOG PS = Eastern Cooperative Oncology Group Performance Status; GDP = growth domestic product; HR = hazard ratio; NRM = nonrelapse mortality; OS = overall survival; Ph = Philadelphia chromosome; WBC = white blood cell count.

Of the 816 patients, 738 (90%) achieved CR after one or two induction cycles. Cumulative incidence of relapse among them was 41% (95% CI: 36-45). In the univariable analysis, factors associated with an increased risk of relapse were older age, higher WBC at diagnosis, Ph-negative ALL, and T-cell phenotype. The only center-, region- and economic-related variable associated with a higher CIR in the multivariable model was a higher number of inhabitants in the city where the treatment center was located hazard ratio (HR) 1.399 (95% CI: 1.084-1.804, *P* = 0.01) (Table 2).

One hundred ten patients died of non-relapsing causes, 49 of them (45%) after allo-HCT. Cumulative incidence of NRM at 5 years was 17% (95% CI: 14-20). In the univariable analysis, older age, higher EOCG PS score at diagnosis, Ph-negative ALL, and diagnosis in the earlier period were associated with higher NRM. In the multivariable adjusted model, none of the center-, region- and economic-related variables were associated with higher NRM (Table 2).

Finally, of the 738 patients achieving CR, 110 died without previous ALL relapse. Of them, causes of death could be identified in 104 (94.5%). Seventy-three patients (70%) died of infectious causes (44 during ALL treatment and 29 after allo-HCT) and were considered to have IRM. Factors associated with higher IRM in the univariable analysis were older age (HR = 1.031 [95% CI: 1.012-1.050], *P* = 0.001) and earlier treatment period (HR = 2.061 [95% CI: 1.289-3.297], *P* = 0.003). Again, none of the center-related and macroeconomic variables were associated with higher IRM (not shown).

The objective of this study was to analyze the potential impact of center-related and macroeconomic variables on the outcome of adult patients with ALL treated within 4 consecutive PETHEMA protocols. These variables were considered along with patient and disease factors to adjust the impact of each of them. Our results indicate that detailed treatment protocols with standardized disease evaluations in reference center laboratories allowed adult patients with ALL to have similar outcomes irrespective of demographic, social and economic characteristics of the centers, cities, and regions where they were treated. These results support the work done by PETHEMA and other National Cooperative Groups in Europe as they assure quality of care and equity to all citizens.

To the best of our knowledge, this is the first study focused on a wide variety of center-related and macroeconomic variables in adult ALL. Contrary to ours, other studies have either focused in one or very few center-related variables (eg, distance to ALL treating center) or have not included well known clinical and genetic factors in their analyses.^16^ Impact of insurance status has also been identified as a prognostic factor for adults with ALL,^17^ although this would not apply to most European public health systems where the great majority of ALL patients are treated in fully accessible public hospitals.

Regarding efficacy, we did not observe differences across centers in terms of center-related and macroeconomic variables. The only variables associated with DFS and OS were those dependent on patient and disease characteristics including age, ECOG performance status and WBC at diagnosis, all of them being previously identified as prognostic factors for ALL patients.^7,11,18^ Noteworthy, none of the variables associated with the experience of the center or size of the city or region where the patient was treated associated with DFS or OS. Patients treated in larger cities had a higher CIR which did not translated into lower OS or DFS. Reasons for this finding are uncertain and might reflect early referrals of more complex cases at diagnosis to larger centers located in highly populated cities.

Center experience has been identified as a prognostic factor in other high-risk procedures performed in hematological patients such as allo-HCT.^19^ However, we did not find any impact of center experience on the outcome of patients. Transplantation is probably a more complex and less well standardized procedure than first-line therapy for ALL, in which physician’s expertise might be more relevant on patient outcomes. Conversely, PETHEMA ALL protocols include very detailed information in terms of dosing and modification of chemotherapeutic agents and transplant indications which might have contributed to mitigate the potential impact of center experience and assure the equity and access to complex therapies to all the population.

Limitations of our study include a local diagnosis of ALL in most patients, a relatively limited sample size and the fact that our results are based on data reported to our cooperative group database. Prospective reporting of data to a cooperative group might indicate certain commitment on scientific studies of these centers. While using data prospectively reported to a cooperative group might harbor a selection bias toward well structured and organized centers, the use of national cancer statistics including all patients diagnosed with ALL in our country would not have allowed us to include homogeneous population of patients treated under the same protocols and with centralized MRD determination, which constitutes a strength of our study.

In summary, center-related and macroeconomic variables do not seem to have an impact on outcomes in newly diagnosed adult patients with ALL in the setting of a public health system with clearly defined and structured treatment protocols. Based on our results, these protocols can be safely administered to ALL patients in all hospitals adhered to a national cooperative ALL group. These data should be considered for nation-wide planning of healthcare infrastructures for cancer patients.

## AUTHOR CONTRIBUTIONS

Conception and Design: PB, JMR. Collection of data: All authors. Data analysis and interpretation: PB, MM, JMR. Manuscript writing: All authors. Final approval of manuscript: All authors.

## DISCLOSURES

PB declares having received honoraria from Allogene, Amgen, BMS, Gilead, Incyte, Jazz Pharmaceuticals, Miltenyi Biomedicine, Novartis and Roche. J.M.R. has received honoraria and grants from Amgen, Pfizer, Incyte, Servier, Takeda and Novartis. All the other authors have no conflicts of interest to disclose.

## SOURCES OF FUNDING

PB received research funding from the Carlos III Health Institute (PI16/01433, PI21/0199), Asociación Española contra el Cáncer (Ideas Semilla 2019) and a PERIS 2018-2020 grant from the Generalitat de Catalunya (BDNS357800). J.M.R. has received grants from Asociacion Española contra el Cáncer (GC16173697BIGA) and HARMONY Alliance (HORIZON 2020, European Union).

## Supplementary Material
