# Supplementary material for: Impact of Center-related Characteristics and Macroeconomic Factors on the Outcome of Adult Patients With Acute Lymphoblastic Leukemia Treated With Pediatric-inspired Protocols
Source: Hemasphere. 2022 Dec 23;7(1):e810. doi: 10.1097/HS9.0000000000000810 (PMC9794204; doi:10.1097/HS9.0000000000000810)
Supplement: Supplementary file 1 [file hs9-7-e810-s001.docx]

**Supplementary table 1. Patient characteristics according to center experience**

| CHARACTERISTIC |  | **Less-experienced centers**  **(≤30 ALL cases reported to PETHEMA)** | **Experienced centers**  **(>30 ALL cases reported to PETHEMA)** | **p value** |
| --- | --- | --- | --- | --- |
| **Age, yr,** median (range) |  | 36 (15 ; 60) | 34 (15 ; 59) | 0.136 |
| **Gender,** n (%) | male | 244 (58) | 231 (59) | 0.689 |
|  | female | 180 (42) | 161 (41) |  |
| **ECOG PS,** n (%) | 0-1 | 327 (82) | 321 (86) | 0.086 |
|  | ≥2 | 73 (18) | 51 (14) |  |
| **WBC, x10^9^/L,** median (range) |  | 13.6 [0 - 842] | 17.2 [0.4 - 393] | 0.342 |
| **CNS infiltration,** n (%) | No | 363 (91) | 344 (93) | 0.208 |
|  | Yes | 37 (9) | 25 (7) |  |
| **Ph+ ALL,** n (%) | No | 365 (86) | 323 (82) | 0.148 |
|  | Yes | 59 (14) | 69 (18) |  |
| **Precursor phenotype,** n (%) | B | 318 (76) | 285 (74) | 0.670 |
|  | T | 102 (24) | 98 (26) |  |
| **Treatment period,** n (%) | 2003-10 | 157 (37) | 184 (47) | 0.004 |
|  | 2011-18 | 267 (63) | 208 (53) |  |

Abbreviations: See footnote of tables 1a and 1b for the definition of abbreviations.

Footnote. Nine centers reported at least than 30 patients with ALL during the study period for a total of 392 patients (48%) reported by these centers.

**Supplementary table 2. Correlation between macroeconomic and center-related variables.**

| **VARIABLES** | **City population** | **Region population** | **Relative Health investment GDP (%)** | **Health investment/inhabitant €** | **GDP per capita (region)** |
| --- | --- | --- | --- | --- | --- |
| **Allo-HCT center** | **0.043** | **0.444** | **0.836** | **0.975** | **0.698** |
| **Number of ALL cases reported** | **0.027** | **0.108** | *0.025* | *0.479* | **0.420** |
| **Number of beds in the hospital** | **0.031** | *0.687* | **0.108** | *0.894* | *0.405* |
| **≥5% Protocol deviations** | **0.221** | *0.718* | **0.672** | *0.957* | **0.508** |

Footnote: p values from Spearman correlations (numeric variables) or median test (categorical and numerical variables). **Bold** font indicates positive Spearman’s rho or greater or equal median while *italics* indicates negative Spearman’s rho or lower median.

Abbreviations: See footnote of tables 1a and 1b for the definition of abbreviations.

**Supplementary table 3. Univariable analysis for clinical, center-related and macroeconomic variables for patient outcomes.**

| **Factor** | | **N** | **OS,**  **HR (95%CI)** | **p** | **N** | **DFS,**  **HR (95%CI)** | **p** | **CIR,**  **HR (95%CI)** | **p** | **NRM, HR (95%CI)** | **p** |
| --- | --- | --- | --- | --- | --- | --- | --- | --- | --- | --- | --- |
| **Age** | | 816 | 1.019 (1.010 – 1.027) | <0.001 | 738 | 1.008 (1.000 – 1.017) | 0.061 | 0.989 (0.979 – 0.999) | 0.029 | 1.039 (1.024 – 1.055) | <0.001 |
| **Gender** | Male | 475 | 1.001 (0.813 – 1.232) | 0.993 | 430 | 1.056 (0.855 – 1.303) | 0.615 | 1.211 (0.938 – 1.564) | 0.140 | 0.808 (0.556 – 1.174) | 0.260 |
|  | Female | 341 | Reference |  | 308 | Reference |  | Reference |  | Reference |  |
| **ECOG PS** | 0-1 | 648 | Reference | 0.003 | 587 | Reference | 0.013 | Reference | 0.440 | Reference | 0.040 |
|  | >1 | 124 | 1.494 (1.147 – 1.945) |  | 109 | 1.421 (1.076 – 1.877) |  | 1.152 (0.807 – 1.645) |  | 1.625 (1.023 – 2.581) |  |
| **WBC** | | 805 | 1.002 (1.001 – 1.003) | <0.001 | 728 | 1.002 (1.011 – 1.003) | <0.001 | 1.003 (1.001 – 1.004) | <0.001 | 0.999 (0.997 – 1.002) | 0.510 |
| **CNS infiltration** | No | 707 | Reference | 0.672 | 643 | Reference | 0.736 | Reference | 0.530 | Reference | 0.700 |
|  | Yes | 62 | 1.094 (0.722 – 1.658) |  | 54 | 1.076 (0.704 – 1.644) |  | 1.166 (0.721 – 1.885) |  | 0.850 (0.374 – 1.933) |  |
| **Ph status** | Negative | 688 | 1.416 (1.042 – 1.924) | 0.026 | 615 | 1.428 (1.056 – 1.931) | 0.021 | 2.244 (1.461 – 3.446) | <0.001 | 0.649 (0.424 – 0.995) | 0.047 |
|  | Positive | 128 | Reference |  | 123 | Reference |  | Reference |  | Reference |  |
| **Cell precursor** | B | 603 | Reference | 0.116 | 552 | Reference | 0.127 | Reference | 0.014 | Reference | 0.260 |
|  | T | 200 | 1.205 (0.955 – 1.521) |  | 175 | 1.204 (0.948 – 1.529) |  | 1.423 (1.073 – 1.888) |  | 0.759 (0.471 – 1.224) |  |
| **Treatment period** | 2003-2010 | 341 | 1.367 (1.111 – 1.683) | 0.003 | 300 | 1.349 (1.071 – 1.699) | 0.011 | 1.144 (0.892 – 1.466) | 0.290 | 1.503 (1.033 – 2.187) | 0.033 |
|  | 2011-2018 | 475 | Reference |  | 438 | Reference |  | Reference |  | Reference |  |
|  | | | | | | | | | | | |
| **Allo HCT center** | No | 292 | Reference | 0.793 | 264 | Reference | 0.453 | Reference | 0.360 | Reference | 0.960 |
|  | Sí | 524 | 1.029 (0.829 – 1.279) |  | 474 | 1.088 (0.872 – 1.358) |  | 1.133 (0.867 – 1.483) |  | 1.009 (0.682 – 1.495) |  |
| **Protocol deviations** | <5% | 445 | 1.150 (0.936 – 1.414) | 0.184 | 401 | 1.144 (0.928 – 1.410) | 0.208 | 0.997 (0.776 – 1.279) | 0.980 | 1.369 (0.935 – 2.004) | 0.110 |
|  | ≥5% | 371 | Reference |  | 337 | Reference |  | Reference |  | Reference |  |
| **Number of ALL cases reported** | <30 | 424 | Reference | 0.540 | 386 | Reference | 0.794 | Reference | 0.550 | Reference | 0.820 |
|  | ≥30 | 392 | 1.066 (0.868 – 1.310) |  | 352 | 1.028 (0.835 – 1.266) |  | 1.080 (0.842 – 1.385) |  | 0.958 (0.659 – 1.392) |  |
| **Number of beds in the hospital** | ≤832 | 404 | 1.073 (0.873 – 1.318) | 0.503 | 368 | 1.134 (0.921 – 1.396) | 0.237 | 1.126 (0.878 – 1.444) | 0.350 | 1.061 (0.731 – 1.540) | 0.760 |
|  | >832 | 412 | Reference |  | 370 | Reference |  | Reference |  | Reference |  |
| **City population** | ≤409,661 | 425 | Reference | 0.703 | 380 | Reference | 0.199 | Reference | 0.014 | Reference | 0.230 |
|  | >409,661 | 391 | 1.041 (0.847 – 1.278) |  | 358 | 1.146 (0.931 – 1.411) |  | 1.367 (1.064 – 1.755) |  | 0.794 (0.546 – 1.154) |  |
| **Region population** | ≤6,578,079 | 459 | Reference | 0.307 | 422 | Reference | 0.396 | Reference | 0.470 | Reference | 0.750 |
|  | >6,578,079 | 357 | 1.114 (0.906 – 1.369) |  | 316 | 1.095 (0.888 – 1.349) |  | 1.096 (0.854 – 1.407) |  | 1.064 (0.731 – 1.548) |  |
| **Relative Health investment GDP (%)** | <6.4 | 366 | 1.130 (0.919 – 1.389) | 0.246 | 328 | 1.081 (0.877 – 1.332) | 0.466 | 0.981 (0.763 – 1.260) | 0.880 | 1.150 (0.792 – 1.672) | 0.460 |
|  | ≥6.4 | 450 | Reference |  | 410 | Reference |  | Reference |  | Reference |  |
| **Health investment/inhabitant €** | <1,312 | 397 | Reference | 0.345 | 366 | Reference | 0.879 | Reference | 0.390 | Reference | 0.250 |
|  | ≥1,312 | 419 | 1.104 (0.899 – 1.356) |  | 372 | 1.016 (0.826 – 1.251) |  | 0.896 (0.698 – 1.149) |  | 1.243 (0.855 – 1.807) |  |
| **GDP per capita region** | ≤ 22,700 | 424 | Reference | 0.450 | 388 | Reference | 0.898 | Reference | 0.700 | Reference | 0.500 |
|  | > 22,700 | 392 | 1.083 (0.881 – 1.330) |  | 350 | 1.014 (0.823 – 1.248) |  | 0.953 (0.742 – 1.222) |  | 1.137 (0.782 – 1.653) |  |

Footnote: See footnote of tables 1a and 1b for the definition of abbreviations.
