# Supplementary material for: Impact of Center-related Characteristics and Macroeconomic Factors on the Outcome of Adult Patients With Acute Lymphoblastic Leukemia Treated With Pediatric-inspired Protocols
Source: Hemasphere. 2022 Dec 23;7(1):e810. doi: 10.1097/HS9.0000000000000810 (PMC9794204; doi:10.1097/HS9.0000000000000810)
Supplement: Supplementary file 2 [file hs9-7-e810-s002.pdf]

# SUPPLEMENTARY FIGURE1

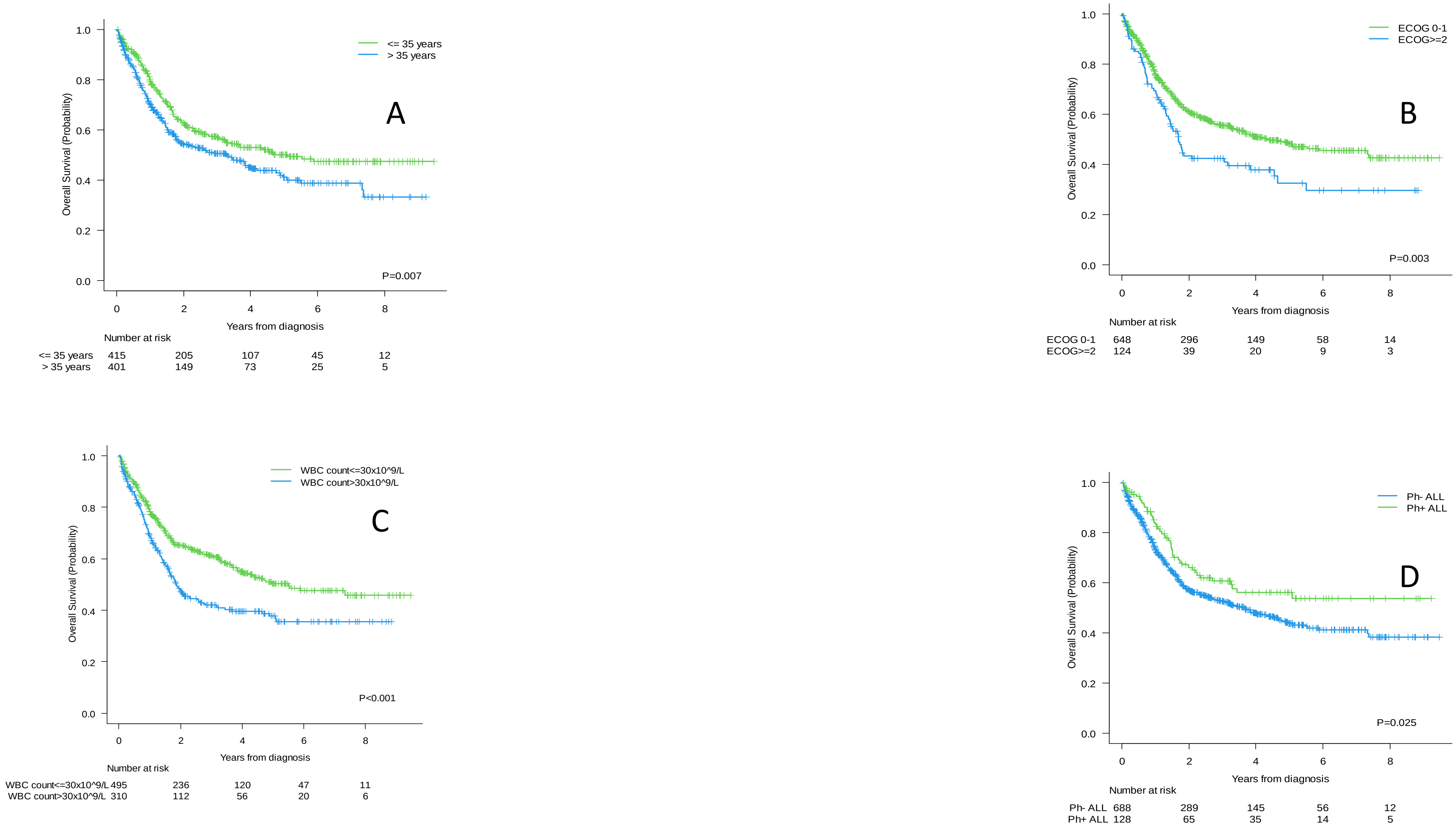


Footnote: OS according to age (A), ECOG performance status (B), WBC (C) and Philadelphia chromosome status (D) at ALL diagnosis.

- **SUPPLEMENTARY FIGURE2**

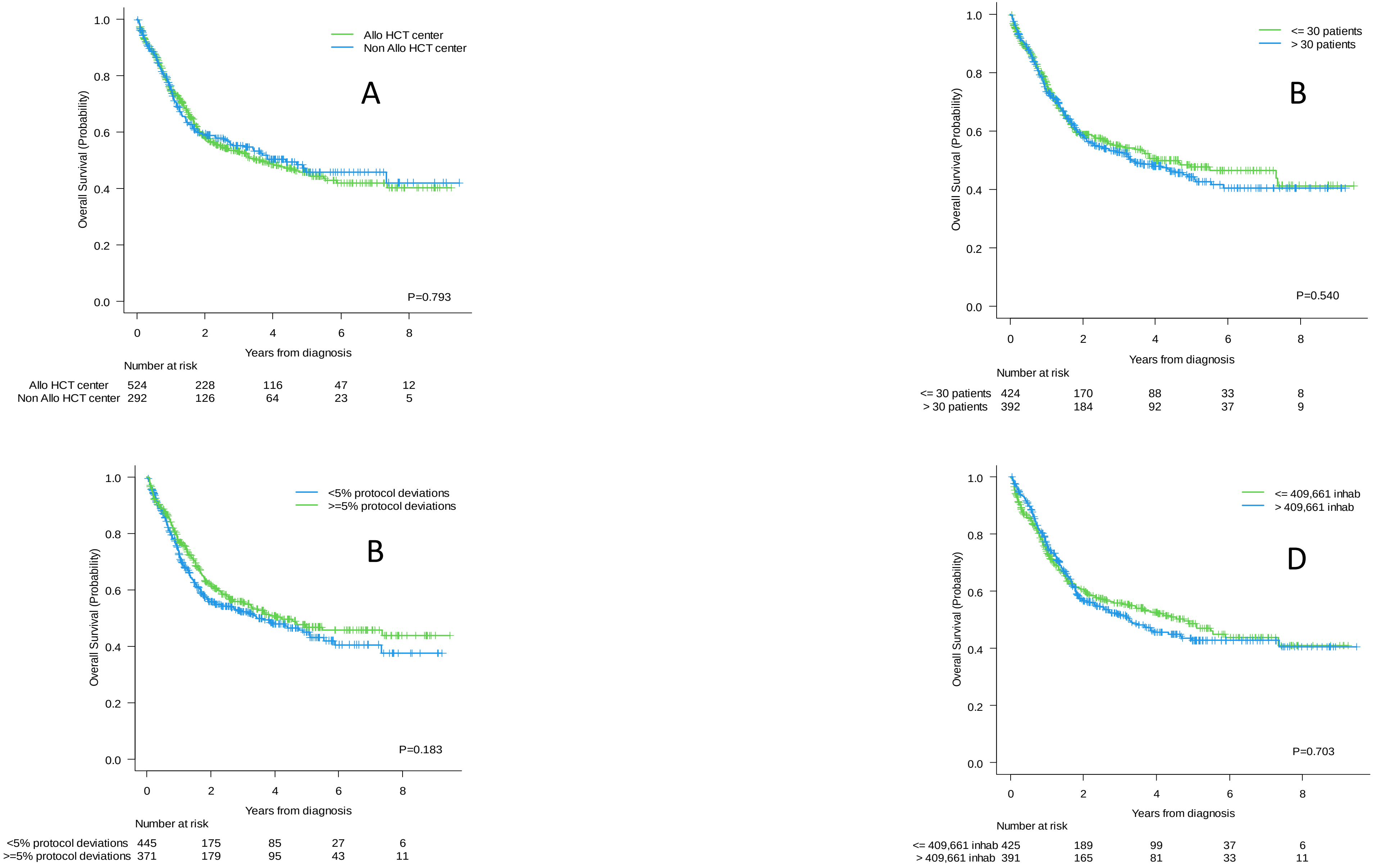


Footnote: OS according to accessibility to HCT in the ALL center (A), number of ALL reported to the PETHEMA database (B), number of major protocol deviations within a center (C) and number of inhabitants in the city where the center is located (D).
